# *QuickStats:* Percentage* of Women Who Have Ever Used Emergency Contraception^^† ^^Among Women Aged 22–49 Years Who Have Ever Had Sexual Intercourse, by Education — National Survey of Family Growth, United States, 2017–2019

**DOI:** 10.15585/mmwr.mm7004a7

**Published:** 2021-01-29

**Figure:**
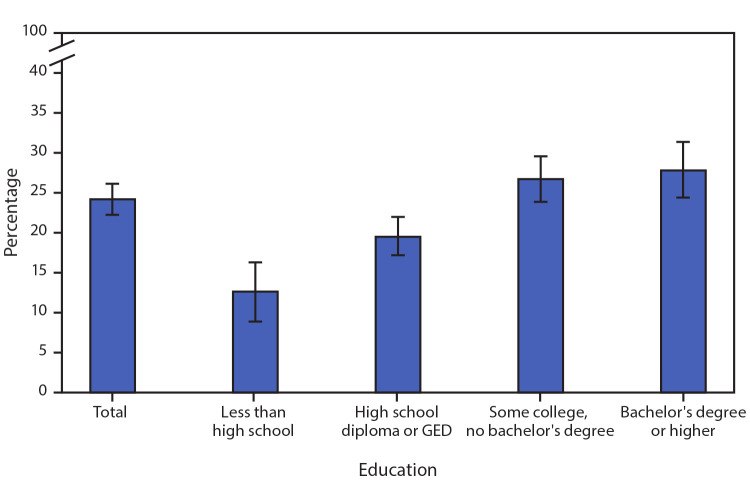
Among women aged 22–49 years who have ever had sexual intercourse, 24.3% have ever used emergency contraception. The percentage of women who have ever used emergency contraception increased with education level, from 12.6% among women without a high school diploma or GED to 27.9% among women with a bachelor’s degree or higher.

**Source: **National Survey of Family Growth, 2017–2019. https://www.cdc.gov/nchs/nsfg/index.htm

